# Persulfate mediated solar photo-Fenton aiming at wastewater treatment plant effluent improvement at neutral PH: emerging contaminant removal, disinfection, and elimination of antibiotic-resistant bacteria

**DOI:** 10.1007/s11356-020-11802-z

**Published:** 2021-01-04

**Authors:** Maria Clara V. M. Starling, Elizângela P. Costa, Felipe A. Souza, Elayne C. Machado, Juliana Calábria de Araujo, Camila C. Amorim

**Affiliations:** Department of Sanitary and Environmental Engineering, Research Group on Environmental Applications of Advanced Oxidative Processes, Universidade Federal de Minas Gerais, UFMG, Presidente Antônio Carlos, 6627, Belo Horizonte, MG 31270-901 Brazil

**Keywords:** Photo-Fenton, Raceway pond reactor, Intermittent iron addition, Persulfate, Antimicrobial resistance

## Abstract

This work investigated an innovative alternative to improve municipal wastewater treatment plant effluent (MWWTP effluent) quality aiming at the removal of contaminants of emerging concern (caffeine, carbendazim, and losartan potassium), and antibiotic-resistant bacteria (ARB), as well as disinfection (*E. coli*). Persulfate was used as an alternative oxidant in the solar photo-Fenton process (solar/Fe/S_2_O_8_^2−^) due to its greater stability in the presence of matrix components. The efficiency of solar/Fe/S_2_O_8_^2−^ at neutral pH using intermittent iron additions is unprecedented in the literature. At first, solar/Fe/S_2_O_8_^2−^ was performed in a solar simulator (30 W m^−2^) leading to more than 60% removal of CECs, and the intermittent iron addition strategy was proved effective. Then, solar/Fe/S_2_O_8_^2−^ and solar/Fe/H_2_O_2_ were compared in semi-pilot scale in a raceway pond reactor (RPR) and a cost analysis was performed. Solar/Fe/S_2_O_8_^2−^ showed higher efficiencies of removal of target CECs (55%), *E. coli* (3 log units), and ARB (3 to 4 log units) within 1.9 kJ L^−1^ of accumulated irradiation compared to solar/Fe/H_2_O_2_ (CECs, 49%; *E. coli*, 2 log units; ARB, 1 to 3 log units in 2.5 kJ L^−1^). None of the treatments generated acute toxicity upon *Allivibrio fischeri*. Lower total cost was obtained using S_2_O_8_^2−^ (0.6 € m^−3^) compared to H_2_O_2_ (1.2 € m^−3^). Therefore, the iron intermittent addition aligned to the use of persulfate is suitable for MWWTP effluent quality improvement at neutral pH.

**Supplementary Information:**

The online version contains supplementary material available at 10.1007/s11356-020-11802-z.

## Introduction

As conventional treatment systems used in municipal wastewater treatment plants (MWWTP) are generally not designed to remove contaminants of emerging concern (CECs), MWWTP effluent constitutes one of the main sources of CECs for environmental waters (Ribeiro et al. 2015). The presence of CECs in surface water (ng–μg L^−1^) may lead to negative impacts to human health as well as to acute and chronic toxicity towards aquatic biota (Barceló 2008). Moreover, CECs have the potential to boost the selection of antibiotic-resistant bacteria (ARB) in natural and engineered environments, such as biological reactors present in MWWTP (Rizzo et al. 2013).

Infections by ARB cause 700,000 deaths annually worldwide (O’Neill 2014), thus being considered one of the main issues regarding public health by the World Health Organization. As ARB are abundant in MWWTP effluent, the implementation of advanced technologies for improving effluent quality has been strongly recommended by environmental and health agencies (Giannakis et al. 2016; Tiedeken et al. 2017). Recent works have evaluated the performance of advanced oxidation processes (AOPs), such as solar photo-Fenton (solar/Fe/H_2_O_2_), on the inactivation of ARB (Giannakis et al. 2018, Giannakis 2017; Michael et al. 2019). AOPs’ effectiveness on ARB removal is related to the formation of highly reactive oxidative radicals (for example: hydroxyl radical, HO^●^), which cause repeated damage to external and internal cell components leading to death (Serna-Galvis et al. 2019a).

Among AOPs, solar photo-Fenton (solar/Fe^2+^/H_2_O_2_) is of particular interest once it assembles the use of renewable energy with an environmentally safe and cheap reagent (iron, Fe^2+^) in the presence of an ordinary oxidant (H_2_O_2_) (Litter et al. 2017; Papić et al. 2009). The regeneration of Fe^3+^ to Fe^2+^ occurs faster under solar irradiation than in dark Fenton reactions. Besides, iron species formed in the system (at acidic pH) may absorb light in the visible range (< 580 nm) leading to an extra pathway for OH^●^ formation. Solar photo-Fenton reached 95% removal of 22 CECs from real MWWTP effluent elsewhere (Klamerth et al. 2013), making this technology especially attractive for post-treatment of this matrix in locations where solar irradiation is abundant (Marcelino et al. 2014).

Among solar reactors used for the application of solar photo-Fenton, the raceway pond reactor (RPR) shows the best cost-benefit when the goal is to remove CEC as it is not necessary to concentrate irradiation for the removal of these contaminants from municipal wastewater treatment plant effluent once they are present in very low concentrations (ng to μg L^−1^). RPR is a low-cost reactor that has been proved successful for the removal of CEC from MWWTP effluent. While costs related to the CPC reactor reach 400 € m^−2^, the cost of a RPR, a reactor that is built with cheap material, is estimated to be around 10 € m^−2^ (Carra et al. 2014; Rivas et al. 2015). Regarding energy power required for wastewater recirculation, CPC uptakes an average of 80 W m^−3^ while RPR employs 4 W m^−3^.

Nevertheless, one of the main limitations of solar photo-Fenton is the acidic pH required for optimum efficiency due to limited iron solubility at neutral pH. As a result, efforts have been made to enable the application of photo-Fenton at neutral pH, such as the use of iron complexing agents (i.e., ferrioxalate, EDDS, citric acid, etc.). However, the addition of complexing agents could increase treatment costs, thus discouraging the application of these technologies mainly in developing countries, suitable locations for the employment of solar technologies due to high levels of incident solar irradiation and lack of infrastructure. As an alternative, the intermittent iron addition strategy may be explored to enable solar photo-Fenton process operation at neutral pH since it guarantees iron availability throughout reaction at higher pH (5–8). Despite successful results obtained using this strategy for the solar/Fe/H_2_O_2_ process (Carra et al. 2013; Clarizia et al. 2017), there are no previous studies investigating its effectiveness using persulfate (S_2_O_8_^2^) as an alternative oxidant.

Considering the scale-up of AOPs using real effluents from MWWTP, real matrix composition represents a challenge for current research, since natural organic matter (NOM) and ions (Cl^−^, HCO_3_^−^, PO_4_^2−^, NO_3_^−^) present in this matrix may act as hydroxyl radical scavengers and cause light attenuation, thus limiting process efficiency (Ribeiro et al. 2019). Hence, the use of alternative oxidants which form selective radicals has gotten attention in the past years. Persulfate emerges as a potential candidate to be used as an alternative oxidant in the photo-Fenton process as it reacts with Fe^2+^ to form sulfate radical (SO_4_^●−^) which has a longer lifespan when compared to OH^●^. Reaction rates between SO_4_^●−^, natural organic matter, and ions are lower when compared to rates reported for OH^●^ (Ahmed et al. 2014; Ahmed and Chiron 2014; Lian et al. 2017; Miralles-Cuevas et al. 2017; Starling et al. 2019b) because SO_4_^●−^ reacts only via electron transfer, while OH^●^ may react via three different mechanisms (OH addition, hydrogen transfer, and electron transfer). Thus, SO_4_^●−^ is more stable for the treatment of real matrices which contain various constituents. In addition, OH^●^ may be formed simultaneously in the presence of SO_4_^●−^ at the natural pH of MWWTP effluent by the reaction of these radicals with hydroxide ions (OH^−^) (Fang et al. 2012; Wang et al. 2017)*.*

Therefore, the aim of this study is to investigate the efficiency of the solar/Fe^2+^/S_2_O_8_^2−^ process at neutral pH using the intermittent iron addition strategy for the removal of three model CECs (caffeine (CAF), carbendazim (CBZ), and losartan potassium (LP)), which are highly consumed and frequently detected in environmental compartments (Starling et al. 2019a, 2019b), from real MWWTP effluent. At first, solar/Fe^2+^/S_2_O_8_^2−^ was applied in a solar simulator. Then, solar/Fe^2+^/S_2_O_8_^2−^ and solar/Fe^2+^/H_2_O_2_ were compared in semi-pilot scale with regard to CECs and ARB removal, disinfection, and acute toxicity. A cost-benefit analysis was also performed to estimate costs associated to each treatment alternative.

## Materials and methods

### Chemicals

LP, CAF, and CBZ were purchased from Sigma-Aldrich. Table S1 (Supplementary Material) shows the chemical structures and physicochemical properties of each CEC. Hydrogen peroxide and sodium persulfate were purchased from MERCK. Sodium metavanadate and ascorbic acid were purchased from Synth. Bovine serum catalase, methanol, and formic acid were purchased from Sigma-Aldrich (≥ 98% purity).

### Sampling

Real MWWTP effluent was sampled after a conventional activated sludge system in a MWWTP located in Belo Horizonte, in the southeast of Brazil, which receives wastewater from 1.5 million inhabitants (290 m^3^ day^−1^), including hospitals, industries, etc. There is no disinfection stage following the biological process in this treatment plant. Real MWWTP effluent was characterized as according to physicochemical parameters as shown in Table S2 (APHA 2012). Samples #1 and #2 were used for experiments performed in laboratory scale, and sample #3 for semi-pilot scale experiments. Acute toxicity was only assessed for sample #3 as this analysis was only performed for experiments conducted in semi-pilot scale (ISO 2007). Real MWWTP effluent samples were spiked with 100 μg L^−1^ of each target CEC (caffeine (CAF), carbendazim (CBZ), and losartan potassium (LP)) prior to experiments in order to enable proper quantification of target compounds in this matrix before and after proposed treatments. Since total inorganic carbon (TIC) in the non-treated effluent samples was below 50 mg L^−1^ (Table S2), a concentration which partially avoids the scavenger effect of bicarbonates (Esteban García et al. 2018), no carbonate removal stage was carried out prior to photo-Fenton reactions conducted at neutral pH.

### Quantification of target CECs in MWWTP effluent

Identification and quantification of CECs spiked to MWWTP effluent were performed using an ultra high-pressure liquid chromatographer (UHPLC; Shimadzu) system connected to a QTOF mass spectrometer (Bruker Daltonics, Impact II). Samples were filtered in 0.22-μm PVDF membranes prior to injection. The UV detector of the UHPLC was set at 240 nm. A C_18_ column (Agilent PoroshellHPH-C18 4.6 × 150 mm, 2.7 μM) was used with the following mobile phases: (A) methanol acidified with 0.1% formic acid and (B) water acidified with 0.1% formic acid at 0.25 mL min^−1^. Sixty percent of A was used until up to 8 min, the rate of A increased to 90% until 10 min, and the run proceeded in this condition until 20 min. Retention times were 6.4 min for CBZ (LOD, 0.58 μg L^−1^; LOQ, 1.94 μg L^−1^), 6.8 min for CAF (LOD, 1.33 μg L^−1^; LOQ, 2.92 μg L^−1^), and 15 min for LP (LOD, 0.71 μg L^−1^; LOQ, 2.38 μg L^−1^). The QTOF mass spectrometer was operated at positive ionization under the following conditions during all runs: capillary 4500 V, nebulizer 0.4 bar, drying gas 5 L min^−1^, and gas temperature 180 °C which enabled the detection of nearly 99% removal for all target compounds by monitoring the following ions: 195, 192, and 423 m/z as shown in Fig. S1.

### Biological assays

Acute toxicity of samples was analyzed using the Microtox® device (Model 500 Analyzer SDI, Azur Environmental), which assesses the sensibility of luminescent marine bacteria *Allivibrio fischeri* exposed to samples (ISO 2007). Luminescence was measured after 5, 15, and 30 min of exposure to non-treated and treated samples in different dilutions, and EC_50_ was obtained by statistical analysis of data performed by using the MicrotoxOmni® Software (81.9% basic test). Results were converted to acute toxicity unit (a.T.u.) as according to Eq. 1. As the highest concentration assessed in the test is 81.9% which corresponds to 1.22 a.T.u., this is the threshold of this analysis. Therefore, only a.T.u. values above 1.22 are considered toxic.

1 $$ \mathrm{a}.\mathrm{T}.\mathrm{u}.=100/{\mathrm{EC}}_{50} $$

Disinfection was assessed by quantifying *Escherichia coli* present in the MWWTP effluent before (10^−6^ NMP/100 mL) (Table S2) and after proposed treatments by using *Colilert* kits from IDEXX (Method 9223 A) as according to manufacturer instructions and the *Standard Methods* (APHA 2012).

The spread plate method was applied for the assessment of ARB inherent to MWWTP effluent and in samples withdrawn (10 mL) after solar/Fe/S_2_O_8_^2−^, solar/Fe/H_2_O_2_, and controls (solar disinfection, Fe/H_2_O_2_, and Fe/S_2_O_8_^2−^). The initial concentration of ARB varied as according to the antibiotic ranging from 10^−2^ to 10^−4^ UFC/100 mL. Quenching agents (catalase or ascorbic acid) were added to samples submitted to oxidative treatments prior to plating for residual oxidant consumption. Plates containing plate count agar (PCA) alone were used for the quantification of total heterotrophic bacteria (THB). For the analysis of ARB, PCA was supplemented with 10 different antibiotics: ampicillin (AMP, 32 mg L^−1^), chloramphenicol (CLO, 32 mg L^−1^), tetracycline (TET, 16 mg L^−1^), erythromycin (ERY, 32 mg L^−1^), amoxicillin (AMO, 32 mg L^−1^), sulfadiazine (INE, 51.2 mg L^−1^), sulfamethoxazole (AZOLE, 35 mg L^−1^), trimethoprim (TRI, 4 mg L^−1^), ciprofloxacin (CIP, 32 mg L^−1^), and trimethoprim + sulfamethoxazole (TRI + AZOLE, 35 mg L^−^1 of each). The concentration and selection of each of these antibiotics were defined as according to the following references (Brooks et al. 2007; Novo et al. 2013; Novo and Manaia 2010; Pei et al. 2006; Yuan et al. 2015). After sample spreading, plates were incubated for 5 days (48 h at 37 °C, followed by 72 h at 27 °C) for colony development and the number of colony-forming units (CFU) was counted in each plate within 48 and 120 h, as according to standard procedures (Brooks et al. 2007; Munir et al. 2011).

### Photo-Fenton treatment at laboratory scale

Solar/Fe^2+^/S_2_O_8_^2−^ assays were conducted in a solar simulator chamber (SUNTEST CPS^+^, ATLAS) equipped with a xenon lamp using the irradiance range set at 268 W m^−2^ (330 to 800 nm) (Fig. S2). S_2_O_8_^2−^ was added as Na_2_S_2_0_8_ (Merck). Experimental conditions tested in this scale are detailed in Table 1. All reactions were conducted in batch and in duplicates in a 400-mL glass recipient placed inside the solar chamber for 60 min. A magnetic stirrer was placed below the solar chamber, and reactions were conducted under continuous stirring (150 rpm) by a magnetic bar placed inside the glass recipient. The original pH of MWWTP effluent, 6.6–7.5 (Table S2), was adjusted to 7 prior to assays #1 to 6. Single and fractioned iron additions were tested for comparison purposes. Assays #7 to 10 were conducted as reference experiments at pH 3 as this is the optimum pH for the operation of Fenton reactions due to increased iron solubility at acidic pH. pH was adjusted by adding HCl to samples (1 mM).

**Table 1:** Experimental conditions tested for the solar/Fe^2+^/S_2_O_8_^2−^ process at neutral pH, reference experiments (pH 3) and controls carried out in a solar chamber

Type	Name	Fe^2+^		S_2_O_8_^2−^		Fe addition	pH
		mg L^−1^	mM	mg L^−1^	mM		
Assay	# 1	2.7	0.05	57.6	0.3	Single	7
	# 2	2.7	0.05	288.2	1.5	Single	
	# 3	27.5	0.5	57.6	0.3	2x (10) + 3x (2.5)	
	# 4	27.5	0.5	288.2	1.5	2x (10) + 3x (2.5)	
	# 5	27.5	0.5	57.6	0.3	Single	
	# 6	27.5	0.5	288.2	1.5	Single	
	# 7	2.7	0.05	57.6	0.3	Single	3
	# 8	2.7	0.05	288.2	1.5	Single	
	# 9	27.5	0.5	57.6	0.3	Single	
	# 10	27.5	0.5	288.2	1.5	Single	
Control	Dark Fenton 1	2.7	0.05	57.6	0.3	Single	7
	Dark Fenton 2	27.5	0.5	288.2	1.5	Single	
	Coagulation 1	2.7	0.05	–	–	Single	
	Coagulation 1	27.5	0.5	–	–	Single	
	Solar only	–	–	–	–	–	
	Solar/S_2_O_8_^2−^	–	–	57.6	0.3	–	
	Solar/S_2_O_8_^2−^	–	–	288.2	1.5	–	

– none

Experiments were performed using two different initial Fe^2+^ concentrations: 2.7 mg L^−1^ (minimum) and 27.5 mg L^−1^ (maximum). Final Fe^2+^ concentrations were always below 5 mg L^−1^ after all experiments. Persulfate concentrations ranged from 57.6 mg L^−1^ (0.3 mM) to 288.2 mg L^−1^ (1.5 mM), which are equivalent to concentrations applied in studies published using solar/Fe^2+^/S_2_O_8_^2−^ for the removal of CECs from MWWTP effluent (Ahmed et al. 2014; Ahmed and Chiron 2014; Miralles-Cuevas et al. 2017). Samples were withdrawn during reactions for the quantification of residual iron (dissolved Fe^2+^, total Fe), residual persulfate (ISO 1998; Liang et al. 2008), CECs, and chemical oxygen demand (COD). Ascorbic acid solution was added to samples for the consumption of residual S_2_O_8_^2−^ (Olmez-Hanci et al. 2014). Control experiments consisted of dark Fenton-like (Fe^2+^/S_2_O_8_^2−^), Fe alone, and solar irradiation alone or with each oxidant (Table 1). Accumulated irradiation per unit of sample volume (*Q*_UV_; kJ L^−1^) during bench scale experiments was calculated as according to Eqs.2 and 3. Constant average incident irradiation UV_G,n_ (W m^−2^) was equivalent to 30 W m^−2^; *Q*_UV,n_ is the energy accumulated from the beginning of the reaction up to each sampling time, Vr (L) is reactor total volume (400 mL), and Ar (m^2^) is the irradiated surface area (Malato et al. 2009).

2 $$ {Q}_{\mathrm{UV},\mathrm{n}}={\mathrm{Q}}_{\mathrm{UV},n-1}+\Delta  t.{\mathrm{UV}}_{\mathrm{G},\mathrm{n}}.\left(\mathrm{Ar}/\mathrm{Vr}\right) $$

3 $$ \Delta  t={t}_n-{t}_{n-1} $$

### Photo-Fenton at semi-pilot scale

Solar/Fe^2+^/S_2_O_8_^2−^ treatment of MWWTP effluent at neutral pH using the intermittent iron addition strategy was also conducted in semi-pilot scale in a raceway pond reactor (RPR) (Fig. S2) located at the Engineering School (UFMG), in Belo Horizonte, Brazil (19° S, 43° W). The RPR used in this study has a maximum volume of 28 L (12 cm liquid depth), and experiments were performed in batch using a total volume of 12 L (5 cm liquid depth). The reactor contains a paddle wheel to provide for sample mixing during reactions. Incident solar irradiation (W m^−2^) was measured throughout reactions by using a global UV radiometer (CUV 5 Kipp&Zonen; 290–400 nm range, 264 mV W^−1^ sensibility), which was positioned horizontally to enable the calculation of accumulated UV irradiation (*Q*_UV_) per volume of sample (L) as according to Eqs.2 and 3 (Malato et al. 2009). Average incident irradiation (UV_G,n_; W m^−2^) during these experiments was calculated considering natural incident irradiation values measured by the radiometer. A reaction in the presence of H_2_O_2_ was also performed under similar conditions for comparison purposes.

MWWTP effluent pH was adjusted to 7 prior to each experiment using HCl (1 mM). 288.2 mg L^−1^ S_2_O_8_^2−^ was used for the solar/Fe/S_2_O_8_^2−^, and the equivalent in moles of H_2_O_2_ was applied for the solar/Fe/H_2_O_2_ process (50 mg L^−1^ of H_2_O_2_). Residual hydrogen peroxide concentration was quantified by the metavanadate method (Nogueira et al. 2005). Fe^2+^ was added intermittently during treatment (0, 5, 10, 15, and 20 min), and the final concentration was equivalent to 55 mg L^−1^ for both systems as according to previous studies (Ahmed et al. 2014). Experiments were performed in duplicates using real MWWTP effluent sample #3 (Table S2). Acute toxicity, disinfection (*E. coli*), and CECs were quantified during treatments. Catalase enzyme (460 mg L^−1^ in phosphate buffer) (Poole 2004) or ascorbic acid solutions were added to samples to interrupt reactions as quenching agents to H_2_O_2_ and S_2_O_8_^2−^, respectively, which do not affect sample acute toxicity (Olmez-Hanci et al. 2014).

### Cost-benefit analysis

Cost-benefit analysis was performed considering conditions tested in the semi-pilot scale. The methodology used for the cost analysis was adapted from Miralles-Cuevas et al. (2017). According to this methodology, the total cost (TC) related to the application of a technology (TC, € m^−3^) refers to (i) amortization cost (AC) of the reactor and other equipment, (ii) operational costs (OC), and total volume to be treated per year (*V*_t_; m^3^) (Eq. 4)

4 $$ \mathrm{TC}\ \left(\text{\EUR}\ {\mathrm{m}}^{-3}\right)=\left(\mathrm{AC}+\mathrm{OC}\right)/{V}_{\mathrm{T}} $$

AC was calculated considering 7.5% of the interest rate (BRASIL 2017) in the investment cost and a plant life-time period equivalent to 20 years. The main cost of solar technologies is related to total irradiated surface area which varies according to accumulated UV energy (*Q*_UVG_). *Q*_UVG_ is calculated in relation to the average incident solar UV irradiation in a location (UV_G_) and to the number of hours of operation in a year (*H*_s_ = 12 h*365). Therefore, the surface area for solar (*S*_S_) irradiated reactors may be calculated as follows (Eqs. 5 and 6):

5 $$ {S}_{\mathrm{S}}=\left({Q}_{\mathrm{UV}\mathrm{G}}\ast {V}_{\mathrm{T}}/{H}_{\mathrm{s}}\ast {\mathrm{UV}}_{\mathrm{G}}\right) $$

where *S*_S_ is the surface area required for the solar system, *Q*_UVG_ (kJ L^−1^) is the amount of accumulated energy required to remove 50% of total CEC in each process, *V*_T_ (L) is the total volume of wastewater treated in a year, *H*_s_ is the total number of hours of operation in a year, and UV_G_ is the average local global irradiation (30 W m^−2^ in Belo Horizonte, MG, Brazil). Fifty percent removal was chosen as the minimum removal rate considering results obtained in semi-pilot scale.

Once surface area is determined, investment costs may be defined as according to Eq. 6, where *C*_b_ corresponds to the cost of the reactor with a surface area *S*_S_. Considering that total cost per area of the RPR reactor is 40 times lower than costs calculated for CPC (Carra et al. 2014), the cost of an RPR with a surface area of 1000 m^2^ (SRPR) is estimated to be 12,090 € (*S*_b_) (Water 2016; Miralles-Cuevas et al. 2017).

6 $$ \mathrm{ICS}={C}_{\mathrm{b}}\ast \left({S}_{\mathrm{S}}/{S}_{\mathrm{b}}\right) $$

Reagent prices and electricity costs detailed in Table S3 were used to calculate operational costs (OC). Costs related to each reagent were calculated considering the dose of each reagent to be applied per cubic meter of MWWTP effluent. In addition, a total volume of 1000 m^3^ day^−1^ was used in calculations.

## Results and discussion

### Solar/Fe/S_2_O_8_^2−^: laboratory scale

**Fig. 1:**
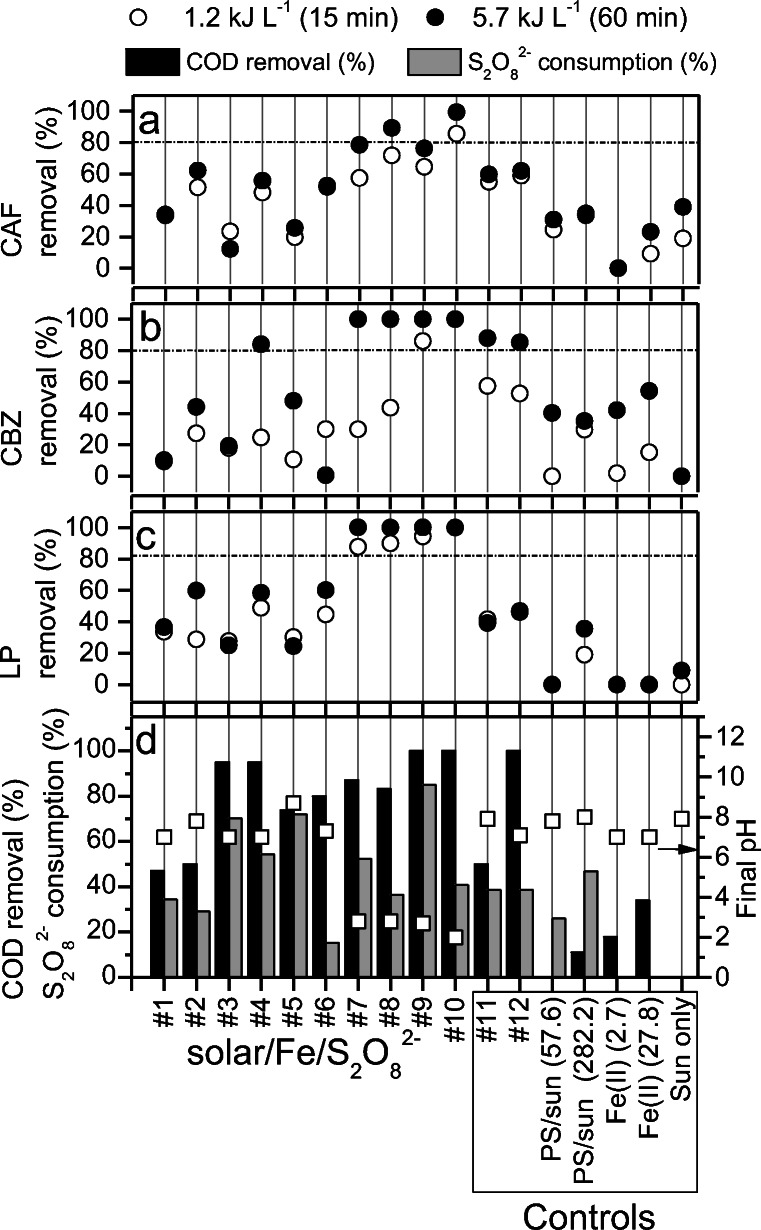
Removal of **a** CAF, **b** CBZ, and **c** LP (III) after 15 (1.2 kJ L^−**1**^) and 60 min (5.7 kJ L^−**1**^) of reaction, and **d** final COD removal, oxidant consumption, and pH via for solar/Fe/S_2_O_8_^2−^experiments at neutral and acidic pH (reference experiments) and controls conducted in a solar simulator. Respective Fe^**2**+^:S_2_O_8_^2−^ concentration (mg L^−**1**^) at pH = 7.0, for single Fe addition: #1 (2.7:57.6), #2 (2.7:288.2), #5 (27.5:57.6), and #6 (27.5:288.2) and for intermittent Fe addition: #3 (27.5:57.6) and #4 (27.5:288.2). At pH = 3.0: #7 (2.7:57.6), #8 (2.7:288.2), #9 (27.5:57.6), and #10 (27.5:288.2). Dark control at pH = 7.0 with single #11 (27.5:288.2) and intermittent Fe addition #12 (27.5:288.2)

Although previous studies have investigated the efficiency of the solar/Fe/S_2_O_8_^2−^ at acidic pH for the removal of CECs (Ahmed and Chiron 2014; Miralles-Cuevas et al. 2017), to this date, there are no works on the application of this treatment at neutral pH using the intermittent iron addition strategy, as proposed in this study. Figure 1 shows removal of CECs (Fig. 1a–c), final pH values, and COD removal (Fig. 1d) obtained after all tested conditions and controls. Dissolved Fe^2+^ concentration and oxidant consumption during assays are shown in Fig. S3 (A, B, C, D).

CAF removal was equivalent to 62% during assay #2 (2.7 mg L^−1^ of Fe^2+^ and 282.2 mg L^−1^ S_2_O_8_^2−^) conducted at neutral pH (Fig. 1a). Meanwhile, Fe/S_2_O_8_^2−^ using 10 times more Fe^2+^ (27 mg L^−1^) reached the same efficiency (61% removal), confirming the occurrence of photo-Fenton reactions instead of dark Fenton alone in the irradiated system, since lower concentrations of reagents were required under irradiation. Solar irradiation alone led to 39% CAF removal (Fig. 1a), indicating indirect photolysis of CAF via radical formation by photolysis of dissolved organic matter and nitrate ions present in the MWWTP effluent, as observed by Wang et al. (2017).

Regarding the removal of CBZ at neutral pH, assay #4 performed with the highest reagent concentrations and intermittent iron additions, as well as its equivalent Fe/S_2_O_8_^2−^ conducted in the dark (Fenton 2) both reached > 80% efficiency compared to a maximum of 60% obtained with a single iron addition (Fig. 1b). These results indicate that continuous availability of iron contributed to appropriate removal of CBZ in these systems. As shown by dissolved Fe^2+^ and S_2_O_8_^2−^ consumption profiles (Fig. S3 D), dissolved Fe^2+^ concentration was low (< 2.5 mg L^−1^) during the entire reaction in assay #4, and no increase was observed after intermittent iron additions. This occurs due to Fe^2+^ conversion to Fe^3+^ after reacting with S_2_O_8_^2−^ as Fe^2+^ concentration remained low in the system even after the addition of this catalyst. Similar observations were made by Carra et al. (2013) when applying this strategy using H_2_O_2_ as an oxidant (Carra et al. 2013), as Fe^2+^ quickly reacted with the oxidant after intermittent additions.

Despite high efficiency observed for CBZ removal by solar/Fe/S_2_O_8_^2−^, control experiments conducted with iron alone led to 50% CBZ removal. This suggests that CBZ may make complexes and precipitate with iron at neutral pH, as it has been observed for CBZ and other CECs (Costa et al. 2019; Norte et al. 2018). This phenomenon may also have occurred in dark Fe/S_2_O_8_^2−^ reactions simultaneously with CBZ degradation by sulfate radicals as 40% of S_2_O_8_^2−^ were consumed during this assay (Fig. S3 D).

When it comes to LP, solar/Fe/S_2_O_8_^2−^ conducted at neutral pH reached 60% removal in one hour (5.7 kJ L^−1^) (assays #2, 4, and 6) (Fig. 1c). LP susceptibility to sulfate radical was also reported in a previous study under UV-C/S_2_O_8_^2−^ (Starling et al. 2019b). Solar/S_2_O_8_^2−^ conducted as a control using the highest concentration of S_2_O_8_^2−^ led to 35% LP (60 min; 5.7 kJ L^−1^) removal and 32% removal of all target CECs (60 min; 5.7 kJ L^−1^). This occurs due to light absorption by S_2_O_8_^2−^ and thermal decomposition of this reagent leading to the formation of sulfate radicals (Ahmed and Chiron 2014).

Higher removal of LP compared to CBZ and CAF may be related to the chemical properties of this compound which contains the highest molar mass (330.739 g mol^−1^) among all compounds along with a greater variety of reactive chemical structures for the attack of the sulfate radical, as shown in Table S1. LP removal within 1.2 kJ L^−1^ (15 min) for all tested solar/Fe^2+^/S_2_O_8_^2−^ conditions was similar to removals obtained after 5.7 kJ L^−1^ (60 min) (Fig. 1). This was not observed for CAF nor CBZ and is probably related to the higher molar absorption coefficient (Table S1) and quantum yield (Starling et al., 2019b) of this compound (Table S1), thus enabling faster degradation when submitted to irradiated processes. Meanwhile, CBZ (191.19 g mol^−1^) and CAF (194.19) share very similar molar mass and chemical structures (Table S1), which explains their similar behavior when submitted to the solar photo-Fenton process. However, as the pKa of CBZ is lower (4.2) than that reported for CAF (10.4) (Carlson et al. 2015), CBZ is probably deprotonated at neutral pH, which explains 40% removal in the presence of iron alone via complexation, as this reagent has positive charge. Expressive removal of CBZ by iron complexation was also observed in a previous study (Costa et al. 2019). Complexation with iron also occurred for CAF, yet to a lower extent (20% removal).

Considering all solar/Fe/S_2_O_8_^2−^ reactions performed at neutral pH, assay #4 conducted using 27.7 mg L^−1^ of Fe^2+^ aligned to the maximum concentration of S_2_O_8_^2−^ was the most favorable for the removal of the sum of CECs (Fig. S4). This assay (65% removal of total CECs) was conducted using the intermittent iron addition strategy and was more efficient than assay #6 (31%) which was performed with similar reagent concentrations yet using a single iron addition. This suggests that the strategy to perform solar photo-Fenton at neutral pH using intermittent iron additions, previously proposed by Carra et al. (2014) for solar/Fe/H_2_O_2_, is also effective when using persulfate as alternative oxidant in this process. Besides, repeated additions of iron in assay #4 led to gradual oxidant consumption in this assay, reaching 54% by the end of the reaction. In contrast, S_2_O_8_^2−^ consumption was slower in the equivalent Fenton reaction reaching a maximum of 40%. This indicates faster Fe^2+^ regeneration from Fe^3+^ under irradiation (Fig. S3 D), as expected for photo-Fenton systems and shown by Eq. 7.

7 $$ \mathrm{Fe}{\left(\mathrm{OH}\right)}^{2+}+{\mathrm{h}\upnu}_{\left(540\hbox{--} 580\ \mathrm{nm}\right)}\to {\mathrm{Fe}}^{2+}+\mathrm{HO}\bullet $$

Dissolved Fe^2+^ concentration was constantly below 1 mg L^−1^ during assay #5 (27.8 mg L^−1^ of Fe^2+^; 56.7 mg L^−1^ of S_2_O_8_^2−^) conducted with a single iron addition (Fig. S3C), as S_2_O_8_^2−^ was promptly consumed in the first five minutes of reaction. Meanwhile, in assay #3 (27.8 mg L^−1^ of Fe^2+^; 56.7 mg L^−1^ of S_2_O_8_^2−^) conducted with intermittent iron additions, Fe^2+^ concentration was stable at nearly 5 mg L^−1^ (Fig. S3C), thus contributing to gradual S_2_O_8_^2−^ consumption throughout treatment. In assays #4 (intermittent additions) and #6 (single addition) which had maximum oxidant concentration (282.2 mg L^−1^), dissolved iron concentration was nearly 2.5 mg L^−1^ throughout the entire reaction (Fig. S3 D) due to higher availability of S_2_O_8_^2−^. As dissolved Fe^2+^ concentration was below 5 mg L^−1^ throughout all assays conducted at neutral pH, there are no risks associated to dissolved iron concentration prior to MWWTP effluent reuse or discharge of treated effluent. Besides, as most of the catalyst added to the system precipitates by the end of the treatment, it may be easily removed.

Higher S_2_O_8_^2−^ consumption (54%) and CEC removal (62%) in assay #4 (intermittent iron additions) compared to assay #6 (single iron addition: 20% oxidant consumption; 35% removal of CECs) confirms the feasibility of using the proposed iron addition strategy for the operation of solar/Fe/S_2_O_8_^2−^ at neutral pH. S_2_O_8_^2−^ consumption in assay #6 was limited due to high turbidity observed right after iron addition, thus limiting further reactions between Fe^2+^ and S_2_O_8_^2−^ and Fe^2+^ regeneration by light absorption (Eq. 7) (Freitas et al. 2017). Consequently, S_2_O_8_^2−^ was in excess in assay #6 contributing to self-scavenger reactions and leading to radical consumption by S_2_O_8_^2−^ and to the regeneration of S_2_O_8_^2−^ as shown in Eqs. 8 and 9 (Kwon et al. 2015).

8 $$ {\mathrm{S}\mathrm{O}4}^{-\bullet }+{\mathrm{S}}_2{{\mathrm{O}}_8}^{2-}\to {{\mathrm{S}\mathrm{O}}_4}^{2-}+{\mathrm{S}}_2{{\mathrm{O}}_8}^{-\bullet } $$

9 $$ {\mathrm{S}\mathrm{O}4}^{-\bullet }+{\mathrm{S}\mathrm{O}4}^{-\bullet}\to {\mathrm{S}}_2{{\mathrm{O}}_8}^{2-} $$

Additionally, no pH decay was observed during solar/Fe/S_2_O_8_^2−^ reactions performed at neutral pH (Fig. 1d). This may be considered an advantage as it is not necessary to adjust the pH prior to reuse or disposal, and there is possible coexistence of SO_4_^•−^ and OH^•^ radicals at neutral pH as shown in Eqs. 10 and 11 (Wacławek et al. 2017).

10 $$ {{\mathrm{SO}}_4}^{-\bullet }+{\mathrm{H}}_2\mathrm{O}\to {\mathrm{H}\mathrm{O}}^{\bullet }+{{\mathrm{H}\mathrm{SO}}_4}^{-} $$

11 $$ {{\mathrm{SO}}_4}^{-\bullet }+{\mathrm{OH}}^{-}\to {{\mathrm{SO}}_4}^{2-}+{\mathrm{HO}}^{\bullet } $$

All solar/Fe/S_2_O_8_^2−^ conditions performed as reference experiments at acidic pH led to > 80% removal of all target CECs (Fig. 1a–c). These results are more satisfactory than those achieved in other studies which also applied persulfate as an alternative oxidant in solar photo-Fenton reactions at acidic pH (Ahmed and Chiron 2014; Miralles-Cuevas et al. 2017), since 92% of all CECs were removed in assay #7 using much lower concentrations of both reagents (2.75 mg L^−1^ of Fe^2+^; 57.6 mg L^−1^ of S_2_O_8_^2^) when compared to the concentration applied in referred studies. Besides, the optimum Fe:S_2_O_8_^2−^ molar ratio obtained for the removal of CECs was 1:6, which is similar to that obtained in Wang et al. (2017), when treating sulfamethazine via UV-Vis LED/Fe(II)/S_2_O_8_^2−^.

Still regarding experiments conducted at pH 3, maximum removal of CECs and COD were obtained in assays #9 (> 90% removal of CECs; > 99% removal of COD) and 10 (> 90% removal of CECs; > 99% removal of COD), both with 27.7 mg L^−1^ of Fe^2+^ in the presence of minimum and maximum S_2_O_8_^2−^ concentrations, respectively (Fig. 1d). S_2_O_8_^2−^ consumption was nearly 85% in assay #9, yet it was limited to 40% in assay #10 (167 mg L^−1^ of remaining S_2_O_8_^2−^), which had nearly five times the initial concentration of S_2_O_8_^2−^ present in assay #9 (Fig. S3 A). pH decayed to values ranging between 2 and 2.8 during assays conducted as reference experiments at acidic pH.

### Solar/Fe^2+^/H_2_O_2_ and solar/Fe^2+^/S_2_O_8_^2−^ at semi-pilot scale

#### Removal of CECs

**Fig. 2:**
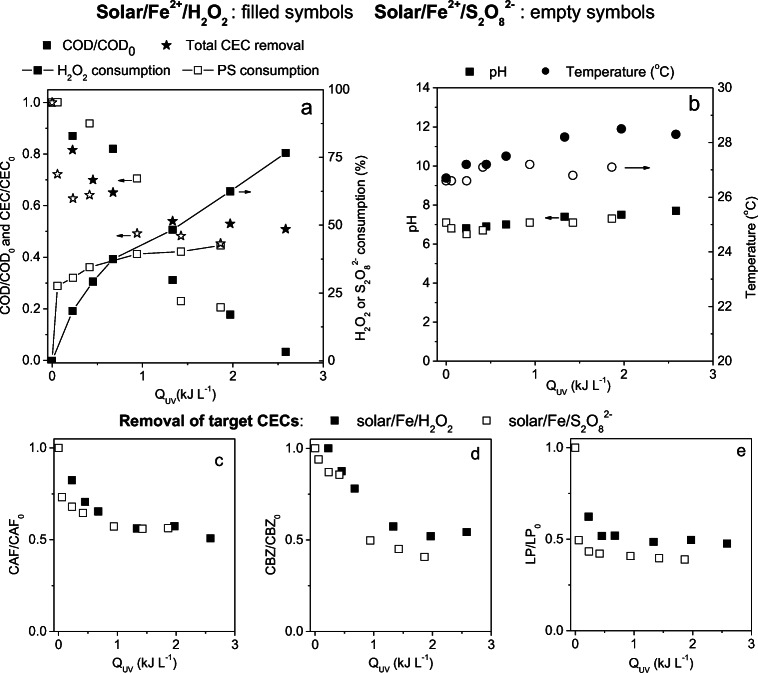
**a** COD (□ scatter), total emerging contaminant (CECs) decay (☆ scatter), and reagent consumption (–■–scatter + line), and **b** pH and temperature monitored during solar/Fe/H_2_O_2_ (filled symbols) and solar/Fe/S_2_O_8_^2−^(empty symbols) conducted at neutral pH in a semi-pilot scale RPR, as well as **c** caffeine, **d** carbendazim, and **e** losartan potassium decay during treatments

Figure 2a shows total removal of CECs, consumption of oxidants, and COD removal, during photo-Fenton treatments performed in semi-pilot scale at neutral pH using the intermittent iron addition strategy. Final removal of CECs obtained during solar/Fe/H_2_O_2_ at near-neutral pH reached 49%, and removal efficiencies obtained for CAF, CBZ, and LP (Fig. 2a, c–e) were 49%, 45%, and 52%, respectively. In contrast, 55% removal of total CECs was obtained (43% CAF, 59% CBZ, and 61% LP) via solar/Fe/S_2_O_8_^2−^ (Fig. 2a, c–e). Removal rates are lower than those obtained by Miralles-Cuevas et al. (2017) and Ahmed and Chiron (2014), who reached more than 90% removal of a mixture of CECs and carbamazepine, respectively. This difference is related to the pH of reactions, as referred studies were conducted at acidic pH.

S_2_O_8_^2−^ consumption was faster in the beginning of the reaction when compared to H_2_O_2_, showing a slower consumption after 15 min of reaction (*Q*_UV_ = 0.5 kJ L^−1^) (Fig. 2a). Meanwhile, H_2_O_2_ consumption increased along the reaction reaching a maximum of 75% (*Q*_UV_ = 2.5 kJ L^−1^). Total decay of CECs followed a similar pattern to that observed for oxidant consumption in solar/Fe/S_2_O_8_^2−^, as it occurred very quickly in the beginning of the reaction and stabilized after 30 min (*Q*_UV_ = 1 kJ L^−1^). In contrast, removal of CECs occurred only until 25 min of reactions (*Q*_UV_ = 1.25 kJ L^−1^) in the solar/Fe/H_2_O_2_, yet H_2_O_2_ consumption continued to occur. This indicates higher selectivity of sulfate radicals towards CECs when compared to hydroxyl radicals. As COD decreased continuously in both treatments, these oxidants are also being consumed by matrix components present in a higher concentration than target CECs in the matrix. CEC removal and COD decay, aligned to oxidant consumption profiles are shown in Fig. 2a, and reveal that oxidant consumption by matrix components is more significant for H_2_O_2_, thus confirming the advantages of using persulfate as an alternative oxidant in the photo-Fenton process for the treatment of real matrices as it is less reactive towards matrix components.

Considering that the same molar concentration of each oxidant was used in both systems, yet lower accumulated irradiation was required for proper removal of CECs during solar/Fe/S_2_O_8_^2−^, this process was more effective for the removal of CECs from real MWWTP effluent at neutral pH when compared to solar/Fe/H_2_O_2_. Carra et al. (2013) observed pH decay after every iron addition due to the hydrolysis of Fe^2+^, when applying solar/Fe/H_2_O_2_ at neutral pH using intermittent iron additions. As shown in Fig. 2b, no pH decay was observed during reactions performed in this study due to matrix buffer effect, confirming results obtained at laboratory scale.

Solar/Fe/S_2_O_8_^2−^ also showed faster removal of each CEC individually. While 43% of CAF removal were achieved within 30 min (*Q*_UV_ = 0.9 kJ L^−1^), this same efficiency was only achieved after 40 min (*Q*_UV_ = 1.5 kJ L^−1^) via solar/Fe/H_2_O_2_ (Fig. 2c). Additionally, final CAF removal via solar/Fe/H_2_O_2_ (49%) was superior to that (20%) reached by Klamerth et al. (2010) under solar irradiation, as he used lower Fe^2+^ concentration.

Among all target compounds, LP was the most sensitive in terms of reaction rate, showing 50% decay within 5 min (*Q*_UV_ = 0.06 kJ L^−1^) when persulfate was used as an oxidant (Fig. 2e). LP removal during this treatment stabilized at nearly 60%, while maximum removal using H_2_O_2_ as an oxidant was 52% (35 min, *Q*_UV_ = 1.3 kJ L^−1^), therefore indicating higher reactivity of LP to sulfate radical–based AOPs when compared to hydroxyl radical, as also observed under UV-C irradiation (Starling et al., 2019a). A higher molar absorption coefficient associated to LP when compared to CAF and CBZ (Table S1) probably led to faster removal of this target compound, as also observed in laboratory scale.

With regard to CBZ (Fig. 2d), while 30 min (*Q*_UV_ = 1 kJ L^−1^) was necessary to remove 50% of CBZ via solar/Fe/S_2_O_8_^2−^, the same removal was only achieved after 45 min (*Q*_UV_ = 2 kJ L^−1^) under solar/Fe/H_2_O_2_ at neutral pH. Interestingly, both processes showed the same degradation profile, with slow removal before 15 min of treatment followed by a fast decay after all iron additions. This confirms the effect of iron complexation towards CBZ removal as shown by control experiments performed in laboratory scale (Fig. 1b).

#### Impact of treatments on acute toxicity and disinfection

**Fig. 3:**
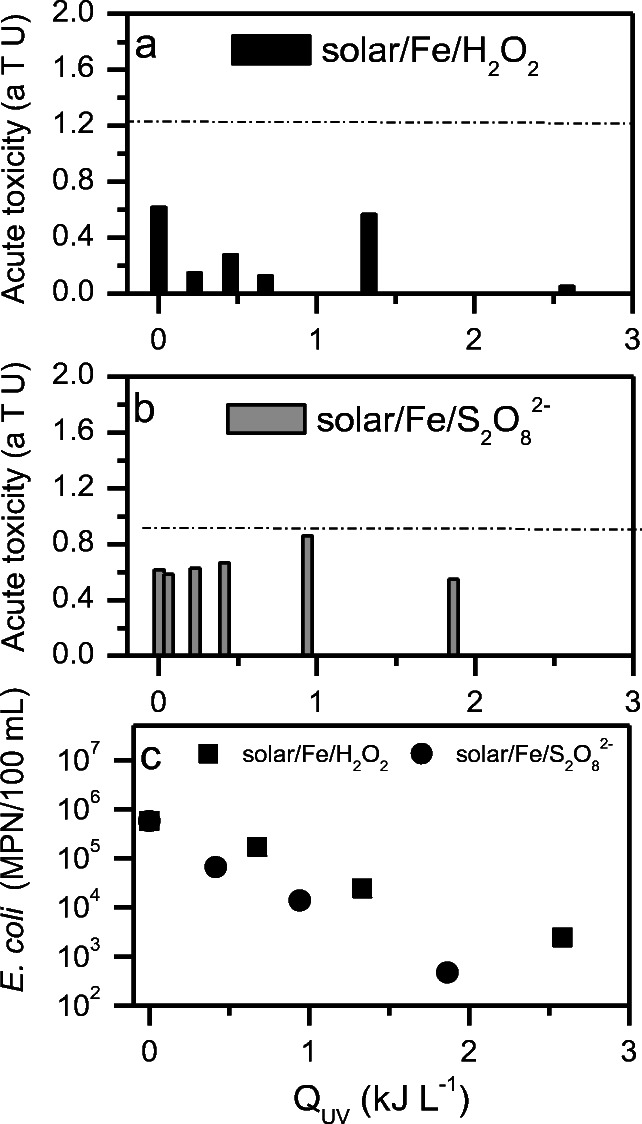
Acute toxicity values obtained for samples withdrawn during **a** solar/Fe/H_2_O_2_ and **b** solar/Fe/S_2_O_8_^2−^ assays conducted at neutral pH in a raceway pond reactor (values above the dashed line are toxic) and **c** *E. coli* decay during solar/Fe/H_2_O_2_ and solar/Fe/S_2_O_8_^2−^ in the same experiment

Acute toxicity assays performed with samples withdrawn during solar/Fe/H_2_O_2_ and solar/Fe/S_2_O_8_^2−^ at neutral pH in the RPR reactor showed that MWWTP effluent was not toxic towards luminescent bacteria (a.T.u. = 0.6) before treatment, and that none of the processes generated toxicity since all values were below 1.21 a.T.u. (line) (Fig. 3a, b) as previously reported elsewhere (Esteban García et al. 2018; Freitas et al. 2017). These results suggest that it would be appropriate to apply any of these processes prior to the disposal of treated effluent.

Bernabeu et al. (2012) investigated acute toxicity of a mixture of CECs (initial concentration = 5 mg L^−1^), including CAF, present in MWWTP effluent upon *Allivibrio fischeri*, before and after treatment, and found an increase in acute toxicity in the beginning of treatment, followed by proper removal after solar/Fe/H_2_O_2_ at neutral pH. This was confirmed by Freitas et al. (2017) who obtained no toxicity for MWWTP effluent containing CECs neither before nor after treatment by solar/Fe/H_2_O_2_ at neutral pH. However, to this date, there are no studies reporting the evolution of acute toxicity during solar/Fe/S_2_O_8_^2−^ treatment of real MWWTP effluent at neutral pH.

When it comes to wastewater reuse, disinfection is a critical endpoint. As observed in Fig. 3c, solar/Fe/S_2_O_8_^2−^ was more effective on the removal of *E. coli* (3 log units; *Q*_UV_ = 1.9 kJ L^−1^) than solar/Fe/H_2_O_2_ (2.5 log units; *Q*_UV_ = 2.5 kJ L^−1^). Results obtained by Esteban García et al. (2018) for *E. coli* removal via solar/Fe/H_2_O_2_ were similar to those observed here for solar/Fe/S_2_O_8_^2−^. Although it is still necessary to investigate mechanisms of cell damage via sulfate radical–based oxidation processes, it is known that external and internal cell photo-Fenton reactions occur via oxidation by hydroxyl radicals (Feng et al. 2020; Xiao et al. 2019).

#### Inactivation of ARB

**Fig. 4:**
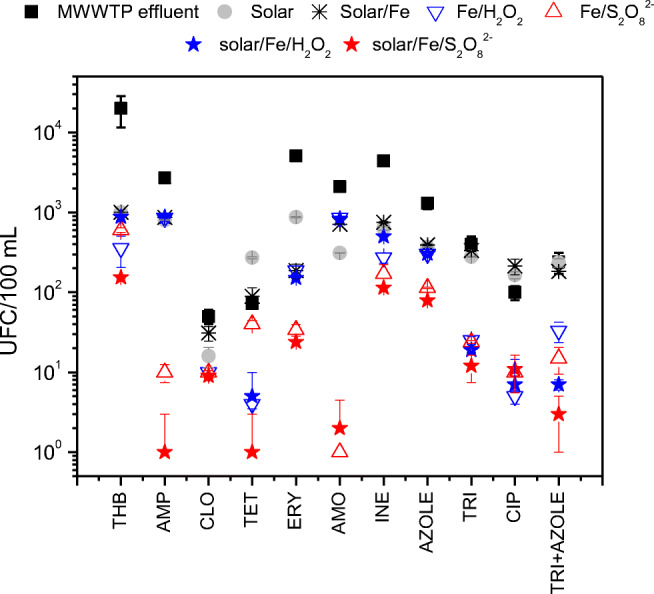
Inactivation of ARB after solar/Fe/S_2_O_8_^2−^ and solar/Fe/H_2_O_2_ compared to non-treated MWWTP secondary effluent. (THB, total heterotrophic bacteria; AMP, ampicillin; CLO, chloramphenicol; TET, tetracycline; ERY, erythromycin; AMO, amoxicillin; INE, sulfadiazine; AZOLE, sulfamethoxazole; TRI, trimethoprim; AZOLE + TRI, sulfamethoxazole + trimethoprim)

Figure 4 reveals that the removal of strains resistant to ampicillin (AMO), chloramphenicol (CLO), erythromycin (ERY), amoxicillin (AMO), sulfadiazine (INE), sulfamethoxazole (AZOLE), and to the combination of trimethoprim and sulfamethoxazole (TRI + AZOLE) was higher via solar/Fe/S_2_O_8_^2−^ compared to solar/Fe/H_2_O_2_, reaching a maximum of 3 log units for bacteria resistant to AMP and AMO. In another study, 6 log decay of carbapenem-resistant bacteria in real hospital wastewater was achieved within 50 min via solar/Fe/S_2_O_8_^2−^ at neutral pH using citric acid as a complexing agent when compared to 3.5 log within 300 min via solar/Fe/H_2_O_2_ (Serna-Galvis et al. 2019b).

Overall, disinfection mechanisms during photo-Fenton processes occur via extra- and intracellular mechanisms. The exposition to UV-A alone leads to increased cell permeability due to stress and induces the formation of reactive oxygen species (ROS), thus culminating in internal photo-Fenton mechanisms which lead to accumulated cell damage followed by death. The period in which ARB accumulate damage was recently nominated as lag period (Serna-Galvis et al. 2019a). Besides the combined detrimental effect of UV light and heat, solar irradiation alone induces a sequence of oxidative outcomes inside cells described as the internal photo-Fenton. Internal photo-Fenton is driven by the loss of Fe^2+^ from enzymes and clusters that contain Fe, leading to intracellular damage with loss of protein function followed by death (Giannakis et al. 2017). The addition of oxidant and iron salts to the matrix accelerates the natural process by enhancing internal and launching external photo-Fenton reactions. Moreover, the UV-B component in solar irradiation is confirmed to have a direct damaging effect upon DNA (Feng et al. 2020). Although pathways of inactivation of biomolecules—carbohydrates, lipids, proteins, and DNA—via OH^•^ have been intensely discussed in the literature, reaction mechanisms between SO_4_•^−^ and these molecules are not yet fully elucidated (Xiao et al. 2019). A recent study suggests that higher inactivation of ARB via sulfate radical–based AOPs may be related to the coexistence of OH• and SO_4_^•−^ radicals in the system at neutral pH (Xiao et al. 2019).

As shown in Fig. 4, removal of total and resistant bacteria via solar disinfection (sun only) was limited to a maximum of one log unit, and solar/Fe/S_2_O_8_^2−^ was 2 to 3 log units more efficient than solar disinfection for both THB and ARB. These results confirm the advantage of solar/Fe/S_2_O_8_^2−^ when compared to solar disinfection alone for the removal of ARB. Although some studies reveal that ARB relative abundance increases during solar photo-Fenton treatment, it may not be necessary to evaluate the ARB inactivation rate as a particular indicator, since it takes longer to remove pathogen indicators as the former are present in higher concentrations in real wastewater when compared to ARB (Fiorentino et al. 2019). Also, solar/Fe/S_2_O_8_^2−^ was similarly effective for total and resistant bacteria (Fig. 4), as it has been reported for solar/Fe/H_2_O_2_ (De la Obra et al. 2019; Giannakis et al. 2018).

Control experiment with Fe/S_2_O_8_^2−^ showed similar efficiency to that of solar/Fe/S_2_O_8_^2−^ for the inactivation of bacteria resistant to ERY, INE, AZOLE, TRI, and CIP, thus confirming the Fenton-like effect on the inactivation of ARB (Fig. 4). This may be an advantage when considering continuous treatment operation in the MWWTP overnight, which was more efficient than operation in batch mode for the removal of bacteria resistant to cephalosporin via solar/Fe/H_2_O_2_ at neutral pH (De la Obra et al. 2019). In addition, Fe/S_2_O_8_^2−^ was more effective than solar/Fe/H_2_O_2_ for the inactivation of bacteria resistant to AMP, ERY, AMO, INE, and AZOLE and TRI + AZOLE confirming higher efficiency of sulfate radical–based AOPs when compared to hydroxyl radical–based AOP on the inactivation of pathogens (Wordofa et al. 2017).

It is worthy to note that treatment effectiveness varied among ARBs to the different antibiotics analyzed in this study (Fiorentino et al. 2019) which is probably related to phenotype differences and intrinsic resistance mechanisms pertaining to each strain (Rodríguez-Chueca et al. 2019b; Sharma et al. 2016). Previous studies have indicated the potential of Fe/S_2_O_8_^2−^ (Ahn et al. 2013) and solar/Fe/S_2_O_8_^2−^ on water disinfection (Rodríguez-Chueca et al. 2019a). Here, we call attention to the potential of the solar/Fe/S_2_O_8_^2−^ process at neutral pH on the disinfection and removal of ARB from MWWTP effluent prior to reuse or discharge.

#### Cost-benefit analysis

Table 2 shows the reactor surface area required for each of the proposed treatments. The solar photo-Fenton-like process using persulfate as an oxidant required 0.9 kJ L^−1^ of accumulated irradiation to reach 50% removal of CEC in neutral pH, while the traditional solar photo-Fenton reached the same removal rate after 1.9 kJ L^−1^ of accumulated irradiation. Hence, the surface area required for the solar/Fe/S_2_O_8_^2−^ process was nearly two times lower than that required for the traditional solar photo-Fenton process.

**Table 2:** Surface area (*S*_S_), investment costs (IC), amortization costs (AC), operating costs (OC), and total costs associated to solar/Fe/S_2_O_8_^2−^ and solar photo-Fenton and solar/Fe/H_2_O_2_ at neutral pH using intermittent iron additions as according to results obtained in semi-pilot scale

Variable	Unit	Solar/Fe/H_2_O_2_	Solar/Fe/S_2_O_8_^2−^
Solar photo-reactor surface area (*S*_S_)			
*Q*_UVG_	kJ L^−1^	1.9	0.9
*V*_t_	L	365,000	365,000
*H*_s_	h	4380	4380
UV_G_	W m^−2^	30	30
Surface area	m^2^	4750	2250
Investment costs (IC)			
Cb	€	12,090	12,090
Sb	m^2^	4750	2250
Sb	m^2^	1000	1000
Total IC	€	57,427.50	27,202.50
Amortization cost (AC)			
IC	€	57,427.50	27,202.50
Interest rate (%)	m^2^	7.5	7.5
Period	year	20	20
Total IC	€ year^−1^	430,706.25	204,018.75
Operating costs (OC)			
H_2_O_2_ 33% (v/v)	€ day^−1^	68.63	none
Na_2_SO_8_		None	3329.96
FeSO_4_·7H_2_O		39.05	39.05
H_2_SO_4_		4.17	4.17
NaOH		None	None
Total OC	€ day^−1^	111.84	3373.18
Total cost (TC)			
AC	€ year^−1^	430,706.25	204,018.75
OC	€ day^−1^	111.84	3373.18
Vt	m^3^	365,000	365,000
TC	€ m^−3^	1.18	0.57

Miralles-Cuevas et al. (2017) obtained surface area values equivalent to 3300 m^2^ for the traditional solar photo-Fenton process and 3200 m^2^ for the solar/Fe/S_2_O_8_^2−^ treatment in a CPC-type reactor at acidic pH. Thus, surface area calculated here for the solar/Fe/S_2_O_8_^2−^ treatment is lower than that obtained in the reference study due to lower average local global incident irradiation in Almeria, Spain (UV_G_ = 18.6 W m^−2^) when compared to the average incident irradiation in Belo Horizonte (UV_G_ = 30 W m^−2^), confirming the high potential of using solar irradiation in tropical locations (Marcelino et al. 2014; Esteban García et al. 2018).

Investment and amortization costs were calculated according to reactor surface area (Table 2). Since the required surface area was lower for solar/Fe/S_2_O_8_^2−^, the investment cost related to this process was also lower than that obtained for solar/Fe/H_2_O_2_. As a consequence, the amortization cost related to the solar/Fe/S_2_O_8_^2−^ is nearly half the cost related to the system using H_2_O_2_. In contrast, amortization costs obtained by Miralles-Cuevas et al. (2017) were slightly lower than the ones obtained here. This is related to the interest rate in each country. If the 6% interest rate applicable to Spain was to be applied in this study, AC would drop to 0.9 and 0.4 € m^−3^ for the traditional and solar photo-Fenton-like treatments, respectively.

Operational costs were calculated for both solar photo-Fenton treatments considering the use of 55 mg L^−1^ of Fe (1 mM) and 50 mg L^−1^ of H_2_O_2_ (1.47 mM) or 282.2 mg L^−1^ of S_2_O_8_^2−^ (1.47 mM). Therefore, the volume of acid used to adjust the pH to 7 was also considered as an operational cost for both treatments. Operational costs were higher for the solar/Fe/S_2_O_8_^2−^ when compared to solar/Fe/H_2_O_2_ (Table 2) due to the cost of sodium persulfate which is 26 times more expensive than hydrogen peroxide (Table S3).

Although operational costs related to the application of solar/Fe/S_2_O_8_^2−^ at neutral pH are increased due to high prices associated to sodium persulfate, the lower surface area required for this process pushed the total cost of the operation to a lower price than that observed for solar/Fe/H_2_O_2_ (Table 2). The TC of solar/Fe/S_2_O_8_^2−^ at near-neutral pH in this study is lower than the cost estimated by Miralles-Cuevas et al. (2017) (0.72 € m^−3^) who conducted this treatment at acidic pH, yet in a more expensive reactor.

Finally, total costs obtained in this study for each of the proposed treatments are competitive when compared to other advanced technologies used for the treatment of CEC in MWWTP effluent. A total cost of 1.1–1.9 € m^−3^, for example, was estimated for the treatment of wastewater containing a mix of pesticides by combined photo-Fenton/membrane bioreactor. Other technologies such as adsorption and reverse osmosis alone may cost from 0.07 to 90 USD mg^−1^ of pollutant and lead to the generation of a solid waste or concentrated solution, respectively, which must be disposed of or treated afterwards (Adeleye et al. 2016). In addition, UV-C-based processes are usually associated to higher operational prices as electric energy is required with costs varying from 0.4 to 1.4 € m^−3^ depending on the volume of wastewater treated (Roccaro et al. 2013).

## Conclusions

Solar/Fe/S_2_O_8_^2−^ performed at laboratory scale using total Fe^2+^ concentration equivalent to 27.7 mg L^−1^ at neutral pH achieved up to 60% removal of total CECs with no pH decay. The treatment using intermittent iron additions was more effective for the removal of CECs than that using a single addition, thus confirming, for the first time, the effectiveness of this strategy using persulfate as an oxidant at neutral pH. Dissolved Fe^2+^ and S_2_O_8_^2−^ consumption profiles showed that dissolved Fe^2+^ concentration was lower than 5 mg L^−1^ during the entire reaction, confirming the safe discharge of this treated effluent.

Solar/Fe/H_2_O_2_ and solar/Fe/S_2_O_8_^2−^ conducted at neutral pH in a raceway pond reactor led to a maximum of 49% (*Q*_UV_ = 2.5 kJ L^−1^) and 54% (*Q*_UV_ = 1.9 kJ L^−1^) removal of total CECs, respectively. Therefore, solar/Fe/S_2_O_8_^2−^ was more effective on the removal of the sum of CECs in real MWWTP effluent reaching a higher removal rate under lower accumulated irradiation (*Q*_UV_). This may be associated to higher selectivity of oxidative radicals formed in the presence of sulfate as an oxidant towards CECs rather than matrix components during the solar photo-Fenton treatment.

In addition, solar/Fe/S_2_O_8_^2−^ was also more effective on the disinfection of MWWTP effluent, removing 3 log units of *E. coli* and ARB, demonstrating the potential of using this treatment prior to MWWTP effluent reuse. As the proposed treatment did not generate acute toxicity upon *Allivibrio fischeri*, it may also be used safely as tertiary treatment prior to discharge. Besides, cost-benefit analysis indicated competitive costs of the proposed solar/Fe/S_2_O_8_^2−^ when compared to solar/Fe/H_2_O_2_ and other technologies which are usually applied as advanced treatment for the improvement of MWWTP effluent quality.

## Supplementary information

ESM 1 (DOCX 1776 kb)
