# Supplementary material for: Persulfate mediated solar photo-Fenton aiming at wastewater treatment plant effluent improvement at neutral PH: emerging contaminant removal, disinfection, and elimination of antibiotic-resistant bacteria
Source: Environ Sci Pollut Res Int. 2021 Jan 4;28(14):17355–68. doi: 10.1007/s11356-020-11802-z (PMC8004486; doi:10.1007/s11356-020-11802-z)
Supplement: Supplementary file 1 — (DOCX 1776 kb) [file 11356_2020_11802_MOESM1_ESM.docx]

PERSULFATE MEDIATED SOLAR PHOTO-FENTON AIMING AT WASTEWATER TREATMENT PLANT EFFLUENT IMPROVEMENT AT NEUTRAL PH: EMERGING CONTAMINANT REMOVAL, DISINFECTION, AND ELIMINATION OF ANTIBIOTIC RESISTANT BACTERIA

*Maria Clara V M Starling^1^; Elizangela Pinheiro da Costa^1^; Felipe Antônio de Souza^1^; Elayne Cristina Machado^1^; Juliana Calábria de Araujo ^1^; Camila Costa de Amorim^1^*^*^

1. Universidade Federal de Minas Gerais, Research Group on Environmental Applications of Advanced Oxidation Processes, 31270-901, Belo Horizonte, Brazil

*Corresponding author: [camila@desa.ufmg.br](mailto:camila@desa.ufmg.br). Universidade Federal de Minas Gerais, Av. Presidente Antônio Carlos 6627, 31270-901, Belo Horizonte, Brazil. Telephone: +55 31 3409-3677, Fax: +55 31 4409-1879.

**Figures:**

Fig. S1 Mass spectrum view of (top) CAF, (center) CBZ and (bottom) LP obtained in the matrix for a sample containing 500 µg L^-1^ of each compound using HPLC connected to QTOF

**Fig. S2** Schematic illustration of the solar chamber (SUNTEST, ATLAS) used in bench scale experiments (left) and RPR reactor (right) used for solar photo-Fenton experiments

**Fig. S3** (A) Fe^2+^ concentration and (B) S_2_O_8_^2-^ consumption (%) obtained during assays 7’, 8’ 9’ and 10’ conducted at acidic pH using the solar photo-Fenton like process. Empty symbols ( □) represent Fe^2+^concentrations and filled symbols (■) represent S_2_O_8_^2-^ consumption; (C) Fe^2+^ concentration and S_2_O_8_^2-^ consumption (%) obtained during assays 3’, 5’ and controls and (D) Fe^2+^ concentration and S_2_O_8_^2-^ consumption (%) obtained during assays 4’, 6’ and controls (Fe^2+^: □ scatter; H_2_O_2_ consumption –■– scatter + line).

**Fig. S4** Removal of the sum of CECs for all Solar/Fe^2+^/S_2_O_8_^2-^ conditions tested at laboratory scale, reference experiments and controls

**Tables:**

**Table S1** - Chemical structure and physicochemical properties of target compounds

**Table S2** Characterization of the MWWTP effluent used in this study

Table S2 Reagent prices used to calculate operational costs

Fig. S1 Mass spectrum view of (top) CAF, (center) CBZ and (bottom) LP obtained in the matrix for a sample containing 500 µg L^-1^ of each compound using HPLC connected to QTOF

Fig. S2 Schematic illustration of the solar chamber (SUNTEST, ATLAS) used in bench scale experiments (left) and RPR reactor (right) used for solar photo-Fenton experiments.

**
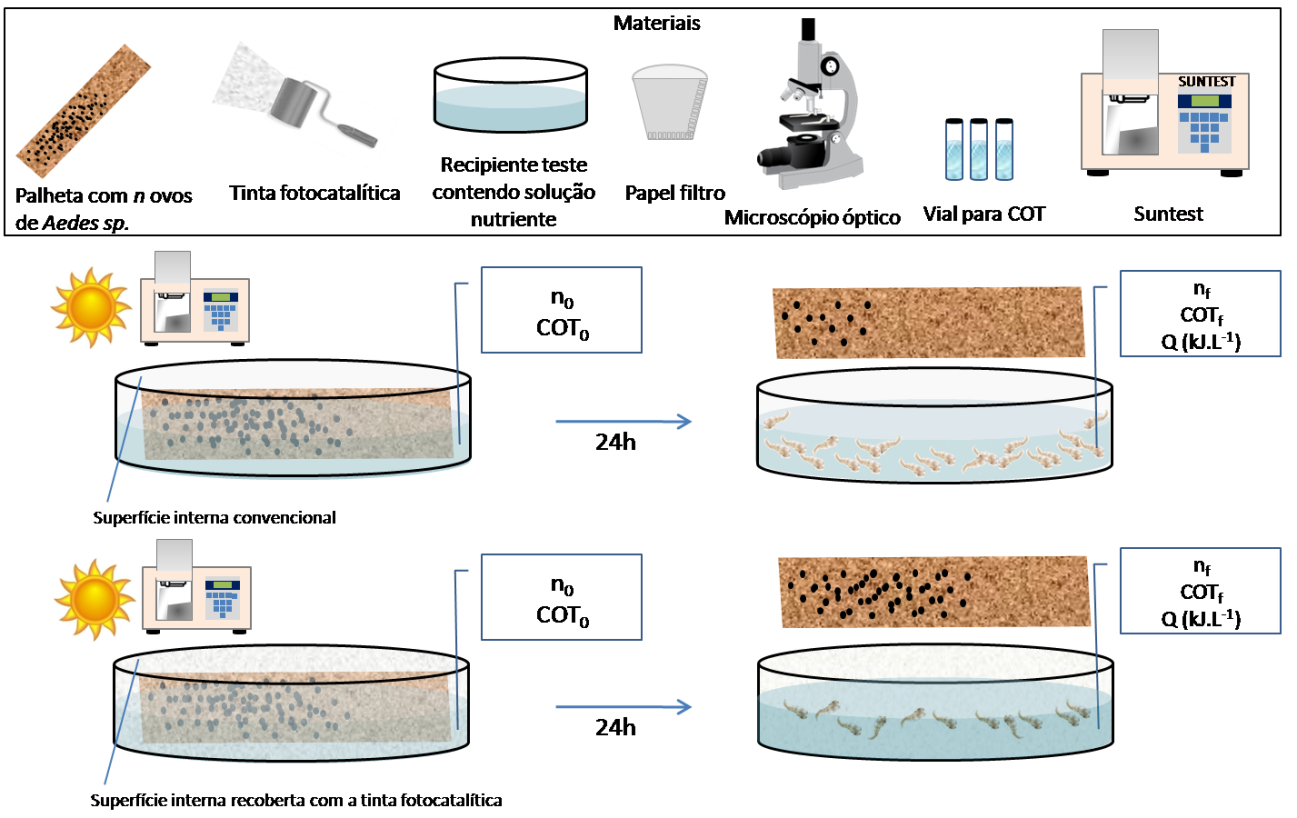
**
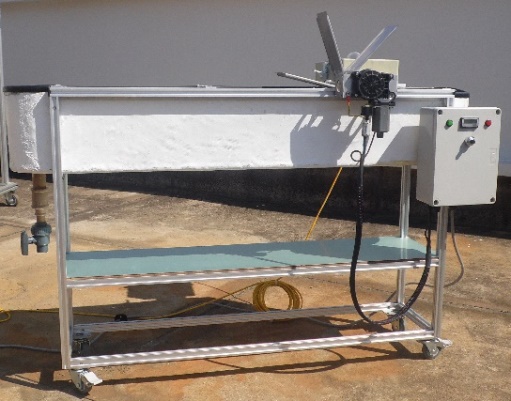


Source: ([COSTA, 2017](#_ENREF_56))

**Fig. S3** (A) Fe^2+^ concentration and (B) S_2_O_8_^2-^ consumption (%) obtained during assays 7’, 8’ 9’ and 10’ conducted at acidic pH using the solar photo-Fenton like process. Empty symbols ( □) represent Fe^2+^ concentrations and filled symbols (■) represent S_2_O_8_^2-^ consumption; (C) Fe^2+^ concentration and S_2_O_8_^2-^ consumption (%) obtained during assays 3’, 5’ and controls and (D) Fe^2+^ concentration and S_2_O_8_^2-^ consumption (%) obtained during assays 4’, 6’ and controls (Fe^2+^: □ scatter; H_2_O_2_ consumption –■– scatter + line).

**Fig. S4** Removal of the sum of CECs for all Solar/Fe^2+^/S_2_O_8_^2-^ conditions tested at laboratory scale, reference experiments and controls

| Compound  **Table S1** Chemical structure and physicochemical properties of target compounds | Abbreviation | Molecular formula | Molar Mass | Chemical structure | pKa | Molar absorption coefficient (ᶓ _254_) |
| --- | --- | --- | --- | --- | --- | --- |
| Unit |  |  | g mol^-1^ |  |  | M^-1^ cm^-1^(pH 7) |
| Losartan Potassium | LP | [C_22_H_22_ClKN_6_O](https://pubchem.ncbi.nlm.nih.gov/search/#collection=compounds&query_type=mf&query=C22H22ClKN6O&sort=mw&sort_dir=asc) | 461.007 | 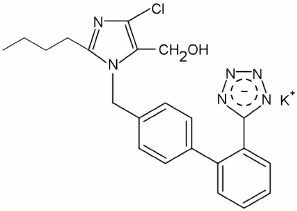 | 4.15 (basic). 14.27 (acidic) ^2^ | 11.772 |
| Caffeine | CAF | C_8_H_10_N_4_O_2_ | 194.194 | 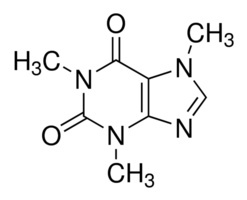 | 10.4^4^  Not observed^5^ | 4.590 |
| Carbendazim | CBZ | C_9_H_9_N_3_O_2_ | 191.19 | 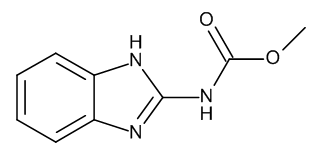 | 4.2 | 3.310 |
| ^1^VCCLAB. Virtual Computational Chemistry Laboratory. http://www.vcclab.org. 2005. ^2^ https://chemaxon.com/consultancy ^3^ Khan SJ. Ongerth JE; Chemosphere 54:355-67 (2004) ^4^ Dean, J., 1999. Lange's Handbook of Chemistry. McGraw Hill Inc., New York, NY, USA.5 J.C. Carlson, M.I. Stefan, J.M. Parnis, C.D. Metcalfe, Direct UV photolysis of selected pharmaceuticals, personal care products and endocrine disruptors in aqueous solution, Water Research 84 (2015) 350-361. | | | | | | |

Table S2 Physicochemical Characterization of the MWWTP effluent used in this study

| ***Parameter*** | ***Unit*** | ***Sample*** | | | | |
| --- | --- | --- | --- | --- | --- | --- |
|  |  | ***1*** | ***2*** | ***3*** | ***Median*** | ***Standard deviation*** |
| pH |  | 6.6 | 7.1 | *7.5* | 7.1 | 0.450924975 |
| Temperature | Celsius | 27.2 | 23 | *23* | 23 | 2.424871131 |
| Total organic Carbon | mg L^-1^ | 14.8 | 10.9 | *-* | 12.85 | 2.757716447 |
| Inorganic Carbon | mg L ^-1^ | 22.3 | 25.3 | *-* | 23.8 | 2.121320344 |
| Chemical Oxygen Demand | mg O L ^-1^ | 158 | 106 | 100 | 106 | 31.89566324 |
| Total Suspended Solids | mg L ^-1^ | 290 | 482 | 360 | 360 | 97.16652373 |
| Volatile Suspended Solids | mg L ^-1^ | 75.5 | 60 | 44 | 60 | 15.75066136 |
| Total Dissolved Solids | mg L ^-1^ | 284.5 | 448 | 328 | 328 | 84.68028106 |
| Volatile Dissolved Solids | mg L ^-1^ | 60.5 | 22 | 18 | 22 | 23.46806341 |
| Conductivity | µS | 427.8 | 769 | 638 | 638 | 172.125187 |
| Hardness | mg CaCO_3_.L ^-1^ | 22.8 | 18.82 | 20.3 | 20.3 | 2.011665976 |
| Hardness | g Mg L^-1^ | 4.9 | 5,64 | 3.4 | 4.15 | 1.060660172 |
| Alkalinity | mg CaCO_3_ L^-1^ | 81.2 | 222.4 | 241.4 | 222.4 | 87.52378724 |
| *E. coli* | NMP/100 mL | - | 7.3E+06 | 2.0E+06 | 4.7E+06 | 3.7E+06 |
| Acute toxicity* | a.T. U. | - | - | 0.62 | 0.62 | 0.12 |
| -: not performed for this sample; *:after spiking. | | | | |  |  |

Table S3 Reagent prices used to calculate operational costs

| **Reagent** | **Price**  **(€ L^-1^ or € kg^-1^)** |
| --- | --- |
| H_2_O_2_ 33% (v/v) | 0.45 |
| Na_2_SO_8_ | 11.8 |
| FeSO_4_.7H_2_O | 0.71 |
| H_2_SO_4_ | 0.1 |
| NaOH | 0.12 |
